# 肺腺癌*EGFR*与*KRAS*基因突变状态分析

**DOI:** 10.3779/j.issn.1009-3419.2015.11.05

**Published:** 2015-11-20

**Authors:** 卉 张, 新杰 杨, 娜 秦, 曦 李, 惠夷 杨, 靖颖 农, 嘉林 吕, 羽华 吴, 权 张, 新勇 张, 敬慧 王, 立娟 周, 树才 张

**Affiliations:** 1 101149 北京，首都医科大学附属北京胸科医院/北京市结核病胸部肿瘤研究所肿瘤内科 Department of Medical Oncology, Beijing Chest Hospital, Capital Medical University/Beijing Tuberculosis and Thoracic Tumor Research Institute, Beijing 101149, China; 2 510663 广州，益善医学检验所 SurExam Clinical Testing Centre, Guangzhou 510663, China; 3 101149 北京，首都医科大学附属北京胸科医院/北京市结核病胸部肿瘤研究所病理科 Department of Pathology, Beijing Chest Hospital, Capital Medical University/Beijing Tuberculosis and Thoracic Tumor Research Institute, Beijing 101149, China

**Keywords:** 肺肿瘤, 表皮生长因子受体, *KRAS*基因, 突变, 腺癌, Lung neoplasms, Epidermal growth factor receptor, *KRAS* gene, Mutation, Adenocarcinoma

## Abstract

**背景与目的:**

表皮生长因子受体(epidermal growth factor receptor, *EGFR*)突变和*KRAS*基因突变是非小细胞肺癌(non-small cell lung cancer, NSCLC)靶向治疗的重要分子标志，与临床治疗疗效密切相关。分析肺腺癌EGFR和*KRAS*基因突变与临床特征的关系。

**方法:**

收集初治肺腺癌患者395例，有可供检测的肿瘤组织标本。利用突变富集液相芯片法进行*EGFR*和*KRAS*基因突变检测。

**结果:**

395例肺腺癌中，*EGFR*基因突变192例(48.9%)，*KRAS*基因突变29例(7.8%)，*EGFR*和*KRAS*基因同时发生突变1例(0.3%)。女性和不吸烟患者*EGFR*基因突变率高于男性和吸烟患者(62.0% *vs* 37.1%, 61.9% *vs* 30.3%)，差异具有统计学意义(*P*＜0.001, *P*＜0.001)；不同年龄、分期及病理取材标本之间差异均无统计学意义(*P*＞0.05)。*KRAS*基因突变在*EGFR*基因野生型患者中发生率(13.5%, 27/200)明显高于*EGFR*基因突变患者(1.0%, 2/192)，差异具有统计学意义(*P*＜0.001)。

**结论:**

肺腺癌*EGFR*基因突变在女性和不吸烟患者中较高，*KRAS*基因突变只在*EGFR*基因野生型患者中较高。在使用TKI靶向治疗药物之前，应同时检测*EGFR*基因和*KRAS*基因的突变情况。

## Abstract (en)

**Background and objective:**

Mutations in epidermal growth factor receptor (*EGFR*) and *KRAS* are important markers in non-small cell lung cancer, which are closely related to the clinical therapeutic effect. To analysis the *EGFR* and *KRAS* gene mutation rate and its relationship with clinical features in patients with lung adenocarcinoma.

**Methods:**

395 patients with treatment naïve lung adenocarcinoma, tumor tissue samples were available for testing. Tumor sample *EGFR* and *KRAS* mutation status were detected using mutant enriched liquidchip.

**Results:**

395 cases of lung adenocarcinoma, *EGFR* mutations were detected in 192 cases (48.9%), *KRAS* mutations were detected in 29 cases (7.8%), and the presence of *EGFR* and *KRAS* mutation were detected in 1 case (0.3%). *EGFR* mutations were found to occur significantly more often in female than in male patients (62.0% *vs* 37.1%, *P* < 0.001) and in never smokers than in smokers (61.9% *vs* 30.3%, *P* < 0.001), no significant differences were observed in age, stage and different biopsy type. *KRAS* mutations were not found to have statistical significance (*P* > 0.05) in each clinical factors, only occurred in the wild type *EGFR* gene in patients (13.5%, 27/200) was significantly higher than that of patients with *EGFR* mutation (1.0%, 2/192), the difference was statistically significant (*P* < 0.001).

**Conclusion:**

In lung adenocarcinomas, *EGFR* mutation was higher in female and non-smoking patients, *KRAS* mutation only in patients with wild-type *EGFR* gene was higher. Before using TKI targeted therapy, *EGFR* and *KRAS* mutations should be detected.

表皮生长因子受体(epidermal growth factor receptor, EGFR)是一种受体型酪氨酸激酶，在许多肿瘤中过表达和(或)发生突变，通过信号转导控制肿瘤生长，并与新生血管生成、肿瘤的侵袭和转移关系密切^[1]^。*KRAS*基因的突变直接启动了EGF通路下游的KRAS途径，并间接启动了EGF信号通路中的PI3K途径，使得非小细胞肺癌(non-small cell lung cancer, NSCLC)进展更迅速，并且使针对EGFR上游通路的药物失效^[2]^。目前多项研究^[3-5]^结果表明存在*EGFR*突变的NSCLC患者应用EGFR酪氨酸激酶抑制剂(tyrosine kinase inhibitors, TKIs)具有较好的临床疗效和安全性，而*KRAS*基因突变也与肺癌治疗的耐药相关。因此，通过对*EGFR*和*KRAS*突变状态的检测，可以筛选出应用EGFR-TKI类药物治疗能够获得更多收益的患者。本研究通过检测分析肺腺癌患者肿瘤组织标本*EGFR*和*KRAS*基因的突变状况，以及其与临床特征之间的关系，为临床指导患者进行有针对性的治疗提供依据。

## 1 材料与方法

### 1.1 材料

收集首都医科大学附属北京胸科医院2006年1月7日-2014年3月21日收治的初治肺腺癌患者395例，所有患者均经细胞学或组织学证实并有可供检测的肿瘤组织标本。其中男性211例，女性184例。年龄20岁-82岁；吸烟(吸烟指数＞20包/年)123例，轻度吸烟(0＜吸烟指数＜20包/年)57例，不吸烟(吸烟指数为0)215例；按2002年美国癌症研究联合会(American Joint Committee on Cancer, AJCC)癌症分期手册(第6版)NSCLC分期标准，Ⅰ期52例，Ⅱ期16例，Ⅲ期64例，Ⅳ期263例(表 1)。

**1:** *EGFR*与*KRAS*基因突变亚组分析 Subgroup analysis of *EGFR* and *KRAS* mutation status

Clinical characteristics	*n*	*EGFR* gene (*n*=394)^*^		*P*	*KRAS* gene (*n*=393)^**^		*P*
		Mutation	Wild type		Mutation	Wild type	
Gender				＜0.001			0.181
Male	211 (53.4%)	78 (37.1%)	132 (62.9%)		19 (9.1%)	190 (90.9%)	
Female	184 (46.6%)	114 (62.0%)	70 (38.0%)		10 (5.4%)	174 (94.6%)	
Age (yr)	20-82 (57.91±0.558)			0.265			0.519
＜65	284 (71.9%)	143 (50.5%)	140 (49.5%)		23 (8.1%)	260 (91.9%)	
≥65	111 (28.1%)	49 (44.1%)	62 (55.9%)		6 (5.5%)	104 (94.5%)	
Smoking history				＜0.001			0.155
Non-smoker	215 (54.4%)	133 (61.9%)	82 (38.1%)		11 (5.1%)	204 (94.9%)	
Light-smoker	57 (14.4%)	22 (38.6%)	35 (61.4%)		5 (8.9%)	51 (91.1%)	
Smoker	123 (31.1%)	37 (30.3%)	85 (69.7%)		13 (10.7%)	109 (89.3%)	
TNM stage				0.070			0.736
Ⅰ	52 (13.2%)	33 (64.7%)	18 (35.3%)		3 (5.8%)	49 (94.2%)	
Ⅱ	16 (4.1%)	7 (43.8%)	9 (56.2%)		2 (12.5%)	14 (87.5%)	
Ⅲ	64 (16.2%)	21 (32.8%)	43 (67.2%)		6 (9.4%)	58 (90.6%)	
Ⅳ	263 (66.6%)	131 (49.8%)	132 (50.2%)		18 (6.9%)	243 (93.1%)	
Biopsy type				0.744			0.954
Incisional	173 (43.8%)	86 (50.0%)	86 (50.0%)		15 (8.7%)	158 (91.3%)	
Bronchoscopic	71 (18.0%)	34 (47.9%)	37 (52.1%)		4 (5.8%)	65 (94.2%)	
Core biopsy	82 (20.8%)	38 (46.3%)	44 (53.7%)		5 (6.1%)	77 (93.9%)	
Localisation	50 (12.7%)	24 (48.0%)	26 (52.0%)		4 (8.0%)	46 (92.0%)	
Pleural	11 (2.8%)	4 (36.4%)	7 (63.6%)		1 (9.1%)	10 (90.9%)	
Cells	7 (1.8%)	5 (71.4%)	2 (28.6%)		0 (0)	7 (100%)	
Mediastinum	1 (0.3%)	1 (100%)	0 (0)		0 (0)	1 (100%)	
^*^One sample not tested for *EGFR* mutations; ^**^Two samples not tested for *KRAS* mutations; EGFR: epidermal growth factor receptor.							

### 1.2 主要仪器与试剂

DNA提取试剂盒(美国Promega公司)；引物、探针合成(广州英韦创津生物科技有限公司)；蛋白酶K(德国Merck公司)；链霉亲和素-藻红蛋白、Taq酶和dNTP(美国Invitrogen公司)；DNA聚合酶(美国Promega公司)；微球和Luminex 200^TM^液相芯片阅读仪(美国Luminex公司)；PCR仪(美国BIO-RAD公司)；Nanodrop 1000分光光度计(美国Thermo Scientific公司)；低温离心机(德国Eppendorf公司)。

### 1.3 突变富集液相芯片法

切取5 μm石蜡包埋标本10张，用DNA提取试剂盒从样本中抽提纯化DNA(具体参照产品说明书)，随后用Nanodrop 1000分光光度计对DNA浓度进行定量；所有样品所提的DNA的量足以用于检测。取纯化后的DNA作为模板进行PCR预扩增，多重PCR反应条件为，94 ℃ 30 s，56 ℃ 30 s，72 ℃ 30 s，共35个循环；预扩增产物进行酶切反应，特异切除野生型，反应条件为37 ℃ 30 min；取酶切产物作为模板进行PCR扩增富集突变型，在96 ℃条件下孵育2 min，反应条件为94 ℃ 30 s，52 ℃ 1 min，74 ℃ 2 min，共40个循环；第二次PCR产物与交联了特异探针的微球杂交，杂交反应在96孔板上进行，将PCR产物、微球混合液和杂交液加入反应管中，95 ℃ 5 min，然后60 ℃杂交15 min，加入链霉亲和素-藻红蛋白，60 ℃反应5 min^[6, 7]^；于Luminex阅读仪上读取数据。突变分析由益善医学检验所完成。

### 1.4 统计学方法

采用SPSS 21.0统计软件进行统计分析，*EGFR*与*KRAS*基因突变状态和临床特征分析采用*χ*^2^检验或*Fisher*’精确检验。以*P*＜0.05为差异有统计学意义。

## 2 结果

### 2.1 突变检测结果

395例肺腺癌*EGFR*基因突变检测中，有1例检测失败；18外显子G719X突变10例(2.5%)；19外显子缺失突变93例(23.5%)；20外显子突变4例(1.0%)；21外显子突变81例(20.5%)；同时存在两种突变4例(1.0%)；野生型202例(51.1%)。*KRAS*基因突变检测中，有2例检测失败；外显子2突变27例(6.8%)；外显子3突变2例(0.5%)；野生型364例(92.2%)。同时存在*EGFR* 19外显子缺失突变合并*KARS*外显子2突变1例(0.3%)(表 2)。

**2:** *EGFR*及*KRAS*基因突变检测结果(*n*=395) Results of *EGFR* and *KRAS* mutation status (*n*=395)

Item	*n*	Proportion (%)
*EGFR* gene		
Exton 18 (G719X)	10	2.5
Exton 19 (19del)	93	23.5
Exton 21	81	20.5
L858R	73	18.5
L861Q	6	1.5
L858R2	2	0.5
Exton 20	4	1.0
T790M	1	0.3
S768I	3	0.7
Combination of two mutations	4	1.0
21L858R+20T790M	1	0.3
18G719+20S768I	1	0.3
19del+21L858R	1	0.3
21L858R+20S768I	1	0.3
Wild type	202	51.1
Failed screening	1	0.3
*KRAS* gene		
Mutation	29	7.3
Exton 2	27	6.8
Exton 3	2	0.5
Wild type	364	92.2
Failed screening	2	0.5
Combination of *EGFR* and *KRAS* mutations	1	0.3

### 2.2 突变与临床特征的关系

女性和不吸烟的患者*EGFR*基因突变率高于男性和吸烟患者，其差异具有统计学意义(*P*＜0.001, *P*＜0.001)；不同年龄、分期及病理取材标本之间差异无统计学意义(*P*＞0.05)。与*EGFR*基因突变不同的是，*KRAS*基因突变在性别、年龄、分期、吸烟史及病理取材方式各亚组分析中都未发现差异具有统计学意义(*P*＞0.05)(表 1)。

### 2.3 *EGFR*与*KRAS*基因突变相关性分析

在*EGFR*和*KRAS*基因都检测成功的392例患者中，*EGFR*基因突变阳性患者中有2例(1.0%)同时发现*KRAS*基因突变，而在*EGFR*基因野生型患者中，有27例(13.5%)发现*KRAS*基因突变。*KRAS*基因突变在*EGFR*基因野生型患者中发生率明显高于*EGFR*基因突变患者，差异具有统计学意义(*P*＜0.001)(表 3)。

**3:** *EGFR*基因与*KRAS*基因突变相关性分析(*n*=392) Analysis of the correlation between *EGFR* and *KRAS* gene mutations (*n*=392)

Group	*KRAS* gene		*P*
	Mutation	Wild type	
*EGFR* gene			
Mutation	2 (1.0%)	190 (99.0%)	＜0.001
Wild type	27 (13.5%)	173 (86.5%)	

## 3 讨论

对于NSCLC患者，选择合理的治疗方案十分重要。美国和中国的肿瘤临床指南均明确指出使用EGFR-TKI治疗前必须进行*EGFR*基因突变检测。KRAS是EGFR信号转导通路下游的一个重要节点。TKI治疗耐药与*KRAS*基因突变以及特定的获得性*EGFR*基因突变(T790M突变)有关^[8]^，所以同时检测*KRAS*基因突变有助于选择最能从TKI治疗中获益的患者。

在美国NSCLC患者中只有约10%有*EGFR*突变，然而在亚洲人中有约30%NSCLC存在*EGFR*基因突变^[3]^。Paez等^[9]^对119例NSCLC患者的*EGFR*突变进行分析后发现，在肺腺癌中*EGFR*基因突变率明显高于其它病理类型。本研究在对395例肺腺癌肿瘤组织标本检测中发现有48.6%患者存在*EGFR*突变，低于之前IPASS研究^[3]^中所报道的59.7%的突变率，但与最近PIONEER研究^[10]^报道的50.2%的*EGFR*基因突变率接近，原因可能是IPASS研究人群为靶向治疗优势人群(女性、腺癌、不吸烟)，而PIONEER研究^[10]^与本研究的研究人群一致，都为确诊肺腺癌的患者。本研究中*EGFR*基因突变率高于之前报道的NSCLC中*EGFR*基因平均突变率，也说明中国人肺腺癌中*EGFR*突变率高于其他病理类型。Paez等^[9]^在进一步的亚组分析中发现，女性和不吸烟的患者*EGFR*基因突变率高于男性和吸烟患者。后续的多项临床研究^[11, 12]^中，重复证实了女性、不吸烟患者中*EGFR*突变率明显高于男性和吸烟患者。本研究中女性、不吸烟患者的*EGFR*基因突变率也明显高于男性、吸烟患者(62.0% *vs* 37.1%，61.9% *vs* 30.3%)，且差异达到统计学意义(*P*＜0.001, *P*＜0.001)，与之前的报道结果一致。

*KRAS*基因突变在非亚洲人群中较为常见。Mao等^[13]^对22篇论著纳入的1, 470例肺癌患者进行系统分析，发现231例(16%)患者存在*KRAS*基因突变，明显高于国内衣素琴等^[14]^报道的5.03%和罗炜等^[15]^报道的3.7%的*KRAS*突变率。本研究仅对肺腺癌患者进行*KRAS*突变检测分析，发现7.3%的患者存在*KRAS*基因突变，明显低于Mao等^[13]^研究中报告的肺腺癌中26%的*KRAS*基因突变率，但与国内相关研究报道^[14, 15]^的4.4%-5.3%的*KRAS*突变率接近。其中差异原因可能是*KRAS*基因突变也与*EGFR*基因突变一样，在不同种族或同一种族不同地区中存在差异。本研究虽然纳入病例数较多，但主要为北京及周边地区患者，具有地域的局限性，因此尚需对多地区样本进行检测分析。目前关于*KRAS*基因突变与患者吸烟史的相关性报道结果尚不一致。有文献^[16]^报道，*KRAS*基因在吸烟患者中更易发生。但一项韩国研究^[17]^中发现*KRAS*基因突变与吸烟无关。本研究中也发现*KRAS*基因突变与是否吸烟并无相关性(*P*＞0.05)。另外，与国内多数报道^[14, 15, 18]^结果一致的是，在对性别和年龄的亚组分析中也未发现*KRAS*基因突变存在差异。

大多数研究^[14]^认为*EGFR*和*KRAS*基因突变是相互排斥的。但在后续的一些研究中也发现*KRAS*基因突变可以在*EGFR*基因突变的情况下发生，但发生率要明显低于*EGFR*基因野生型患者。本研究中检测到1例患者同时存在EGFR 19外显子缺失突变和KRAS 2外显子突变，但临床应用TKI药物治疗，疗效不佳。将*EGFR*和*KRAS*基因突变相关性进行分析发现，*EGFR*基因突变阳性的患者中*KRAS*基因突变率为1.0%，而*EGFR*基因野生型患者中*KRAS*基因突变率为13.5%，两者间差异具有统计学意义(*P*＜0.001)。也就是说，*KRAS*基因突变状态与*EGFR*基因突变状态有关，在*EGFR*基因野生型患者中发生率更高^[19]^。

目前尚无任何指南对NSCLC患者的*EGFR*及*KRAS*基因突变检测方法及检测标本做出明确规定。但大型国际多中心临床研究多采用ARMS法进行突变检测^[2-4]^。本研究中所采用新型的突变富集液相芯片法，灵敏度可达到0.1%^[20]^，检测标本不仅包含手术切除的巨大肿瘤组织标本(173/395)，也有支气管镜活检、肺穿刺、胸膜活检、胸水包埋等临床常见的微小组织标本(222/395)。结果仅有1例手术标本和2例支气管镜活检标本因石蜡切片中DNA断裂，导致基因突变检测失败。但各类组织标本包括胸水肿瘤细胞沉渣都可以满足突变富集液相芯片法检测*EGFR*和*KRAS*基因突变的要求，甚至我们在血浆游离DNA的基因突变检测中也取得了满意的结果^[21]^。

肺癌的靶向治疗时代已经来临。初治*EGFR*基因突变的NSCLC患者中TKI治疗有效率虽然达到70%-80%，但还有20%-30%的患者可能因为各种原因存在原发耐药，其中*KRAS*基因突变就是最常见的原因。因此，在NSCLC患者进行TKI药物治疗前进行*EGFR*和*KRAS*基因突变检测是非常必要的，必将成为临床的常规诊断项目。

## References

[1] Rosell R, Carcereny E, Gervais R (2012). Erlotinib versus standard chemotherapy as first-line treatment for European patients with advanced *EGFR* mutation-positive non-small cell lung cancer (EURTAC): a multicenter, open-label, randomized phase 3 trial. Lancet Oncol.

[2] Boram L, Boin L, Gangmin H (2014). *KRAS* mutation detection in non-small cell lung cancer using a peptide nucletic acid-mediated polymerase chain reaction clamping method and comparative validation with next-generation sequencing. Korean J Pathol.

[3] Mok TS, Wu YL, Thongprasert S (2009). Gefitinib or carboplatin-paclitaxel in pulmonary adenocarcinoma. N Engl J Med.

[4] Dahabreh IJ, Linardou H, Siannis F (2010). Somatic *EGFR* mutation and gene copy gain as predictive biomarkers for response to tyrosine kinase inhibitors in non-small cell lung cancer. Clin Cancer Res.

[5] Zhou C, Wu YL, Chen G (2011). Erlotinib versus chemotherapy as first-line treatment for patients with advanced *EGFR* mutation-positive non-small-cell lung cancer (OPTIMAL, CTONG-0802): a multicenter, open-label, randomized, phase 3 study. Lancet Oncol.

[6] Jiang M, Zhang C, Wang J (2011). Adenosine A(2A)R modulates cardiovascular function by activating ERK1/2 signal in the rostral ventrolateral medulla of acute myocardial ischemic rats. Life Sci.

[7] Koo JW, Quintanilla-Dieck L, Jiang M (2015). Endotoxemia-mediated inflammation potentiates aminoglycoside-induced ototoxicity. Sci Translat Med.

[8] Xu QY, Mou HB, Xu N (2012). Mechanism and treatment associated with resistance to EGFR-TKI. Guoji Zhong Liu Xue Za Zhi.

[9] Paez JG, Janne PA, Lee JC (2004). *EGFR* mutation in lung cancer: correlation with clinical response to gefitinib therapy. Science.

[10] Shi Y, Au JS, Thongprasert S (2014). A prospective, molecular epidemiology study of *EGFR* mutations in Asian patients with advanced non-small cell lung cancer of adenocarcinoma histology(PIONEER). J Thorac Oncol.

[11] Ren RX, Li JY, Li XF (2012). Detection of epidermal growth factor receptor mutations in small specimens of non-small cell lung cancer by amplification refractory mutation system. Zhong Liu.

[12] Wang F, Fu S, Tang T (2011). Relationship between mutations of epidermal growth factor receptor gene and clinicopathologic features of non-small cell lung cancers. Zhonghua Bing Li Xue Za Zhi.

[13] Mao C, Qiu LX, Liao RY (2011). *KRAS* mutations and resistance to EGFR-TKIs treatment in patients with non-small cell lung cancer: a *meta*-analysis of 22 studies. Lung Cancer.

[14] Yi SQ, Zhuang Y, Zhu WD (2013). Analysis of *KRAS* gene mutations in non-small cell lung cancer. Zhonghua Lin Chuang Yi Shi Za Zhi (Electronic Edition).

[15] Luo W, Wang H, Xu WJ (2014). Analysis of *KRAS* mutation in patients with non-small cell lung cancer. Guangdong Yi Xue.

[16] Boch C, Kollmeier J, Roth A (2013). The frequency of *EGFR* and *KRAS* mutations in non-small cell lung cancer (NSCLC): routine screening data for central Europe from a cohort study. BMJ Open.

[17] Kim HR, Ahn JR, Lee JG (2013). The impact of cigarette smoking on the frequency of and qualitative differences in *KRAS* mutations in Korean patients with lung adenocarcinoma. Yonesi Med J.

[18] Gao J, Chen JQ, ZHANG L (2012). Relationship between *EGFR* and *KRAS* mutations and prognosis in Chinese patients with non-small cell lung cancer: a mutation analysis with read-time polymerase chain reaction using scorpion amplification refractory mutation system. Zhonghua Bing Li Xue Za Zhi.

[19] Cheng L, Alexander RE, Maclennan GT (2012). Molecular pathology of lung cancer: key to personalized medicine. Mod Pathol.

[20] Wu S, Zhu Z, He J (2010). A novel mutantenriched liquidchip technology for the qualitative detection of somatic mutations in *KRAS* gene from both serum and tissue samples. Clin Chem Lab Med.

[21] Zhang H, Liu D, Zhang SC (2013). Comparison of EGFR signaling pathway somatic DNA mutations derived from peripheral blood and corresponding tumor tissue of patients with advanced non-small-cell lung cancer using liquidchip technology. J Mol Diagn.

